# Adhesion Performance of Rubber Modified Asphalt in Chip Seal: A Molecular Dynamic Study

**DOI:** 10.3390/ma16186324

**Published:** 2023-09-21

**Authors:** Meng Wu, Zhanping You, Dongzhao Jin

**Affiliations:** Department of Civil, Environmental and Geospatial Engineering, Michigan Technological University, 1400 Townsend Drive, Houghton, MI 49931-1295, USA; mewu@mtu.edu (M.W.); dongj@mtu.edu (D.J.)

**Keywords:** rubber-modified asphalt, chip seal, molecular dynamics simulation, nanoindentation, adhesion performance

## Abstract

Chip seals are widely used for asphalt pavement maintenance, yet the understanding of the interaction between asphalt and aggregates embedded in the asphalt layer remains limited. This paper aims to quantify the interaction between asphalt and aggregate at the microscope level to better understand their adhesion performance in chip seals. Rubber-modified and neat asphalt models are established and verified based on various parameters, including density, viscosity, solubility, glass-transition temperature (Tg), and cohesive energy density (CED). Subsequently, nanoindentation simulation is employed to analyze the adhesion force and interface stress between aggregates and asphalt, considering different embedded depths and pull-off speeds. The adhesion energy between asphalt and silica is also calculated. The results indicate that rubber-modified asphalt exhibits lower density, CED, solubility parameters, and Tg while having higher viscosity than neat asphalt. The adhesion force and interface stress display a quadratic relationship with embedded depths and pull-off speeds. Furthermore, the bond between rubber-modified asphalt and silica is stronger than that between neat asphalt and silica. These findings advance the comprehension of asphalt–aggregate adhesion in chip seals and offer insights for optimizing chip seal design through molecular simulation, thereby potentially enhancing asphalt pavement performance.

## 1. Introduction

As the predominant form of pavement, asphalt pavement constitutes over 94% of the road network in the United States [1]. Over time, asphalt pavement is subject to degradation caused by various factors, including exposure to ultraviolet rays, water, and heavy loads. Such degradation manifests in surface cracks, asphalt material aging, rutting, and other issues [2]. Consequently, ongoing maintenance is essential to ensure the pavement’s continued functionality.

In addressing initial pavement distress, various treatment methods are commonly employed, including fog seal, slurry seal, crack sealing, and chip seal [2]. Among these options, chip seal has gained widespread usage due to its ability to address most initial surface distresses in asphalt pavement while offering a compelling degree of cost-effectiveness [3,4,5]. The two primary types of chip seal applications are emulsified asphalt [3] and rubber-modified asphalt [6]. Notably, the latest approach involves incorporating rubber into emulsified asphalt and utilizing it for chip seal applications [7].

Existing research papers employ a range of tests, such as the asphalt bond strength (BBS) test, the rheological test using a dynamic shear rheometer, and the interface bond test (IBT), to evaluate the performance of diverse asphalt materials in chip seals [3,6,7]. These investigations aim to derive improved design solutions for chip seal performance, encompassing aspects such as asphalt modification and aggregate grading. As a result of these studies, a general chip seal performance evaluation system has been developed. However, they do not specifically address the interaction patterns between asphalt and aggregate within the chip seal. During chip seal construction, the aggregate chips are uniformly spread over the surface after applying asphalt emulsion, resulting in the aggregate being embedded within the asphalt layer. The selection of the asphalt type and the depth of embedding are critical aspects of chip seal application, with adhesion performance being a crucial evaluation criterion.

A microscopic mechanical analysis is essential to gain insights into the intricate interaction between asphalt and aggregate in chip seals. For this purpose, atomic force microscopy (AFM) and nanoindentation emerge as two well-suited methods capable of testing the adhesion between solid surfaces [8,9]. However, careful deliberation on experimental sample preparation and cost consumption becomes imperative when aiming to obtain multiple data sets, such as dozens of sets involving considerations of the continuous depth of aggregate embedded in the asphalt layer. Consequently, it is not uncommon for most research papers to limit the number of samples due to these practical constraints [3,4,10].

To overcome the limitations of traditional experimental methods in studying the interaction between asphalt and aggregate in chip seals, this paper proposes the utilization of numerical simulation techniques. Numerical simulation offers the advantage of obtaining multiple data results simultaneously through parallel computing. In the field of asphalt research, common numerical simulation methods include finite elements, discrete elements, and molecular simulation. Among these methods, molecular simulation stands out as a favorable approach due to its ability to elucidate material properties from a molecular perspective and calculate parameters related to energy, mechanics, and thermodynamics, presenting significant advantages [11,12,13]. Notably, molecular simulation can distinctly differentiate material compositions, a capability unavailable in finite element and discrete element methods.

The presence of embedded aggregate within the chip seal and its adhesion to the asphalt layer are critical factors influencing the chip seal’s performance. To comprehensively investigate this crucial interaction, molecular dynamics (MD) is employed in this study to simulate the nano-indentation mechanical behavior of the aggregate on the asphalt surface and calculate the adhesion energy of the aggregate–asphalt interface. The primary objective is to gain deeper insights into how the aggregate and asphalt interact under varying conditions. In this analysis, two types of asphalt are considered: rubber-modified asphalt and neat asphalt. This research aims to shed light on the underlying mechanisms governing the interfacial behavior between the aggregate and asphalt, providing valuable knowledge for designing and optimizing chip seals under different scenarios.

## 2. Methodology

### 2.1. Molecular Model for Rubber-Modified Asphalt

The consensus among scholars categorizes asphalt into four main components: asphaltene, aromatic, saturated, and resin [14]. Asphaltene and resin are commonly regarded as polar components, while aromatic and saturate are non-polar components. The construction of the asphalt molecular model is informed by this understanding. Over time, the evolution of the asphalt molecular model has progressed from the average molecular model to the three-component asphalt molecular model and finally to the widely accepted and widely used four-component asphalt molecular model [12,13].

**Figure 1:**
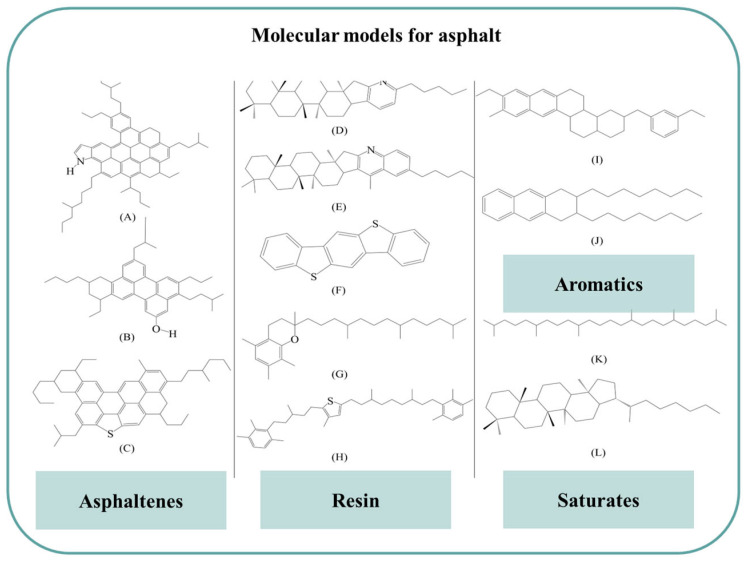
Molecular structure for asphalt [12]. The molecule from (**A**–**L**) corresponds to asphaltene-pyrrole (**A**), asphaltene-phenol (**B**), asphaltene-thiophene (**C**), pyridinohopane (**D**), quinolinohopane (**E**), benzobisbenzothiophene (**F**), trimethylbenzeneoxane (**G**), thioisorenieratane (**H**), PHPN (**I**), DOCHN (**J**), squalene (**K**), hopane (**L**), respectively.

In this study, the four-component asphalt molecular model proposed by Greenfield [12] is employed, as depicted in Figure 1. This model offers a comprehensive representation of the intricate molecular structure of asphalt, facilitating precise simulations and in-depth analyses of its behavior and properties.

**Figure 2:**
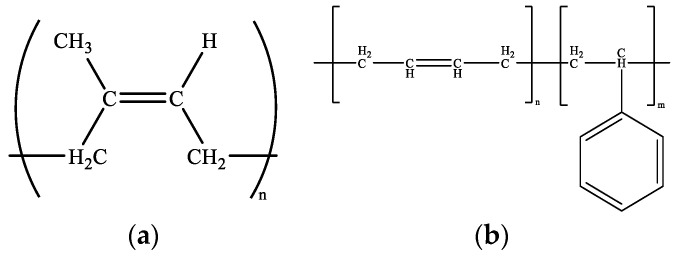
Molecular structure for rubber [15], (**a**) NR, (**b**) SBR.

In this study, the modeling process involves creating a molecular model of rubber-modified asphalt by combining the molecular model of asphalt with the molecular model of rubber. Notably, the current consensus among scholars [10] suggests that, in its initial state, no chemical reaction occurs between rubber and asphalt, though this may change over time due to aging effects. Rubber is characterized as a mixture, with its primary constituents being natural rubber (NR) and styrene-butadiene rubber (SBR). The molecular structure of NR consists of polyisoprene, while SBR exhibits a copolymer structure composed of styrene and butadiene units [15,16]. Figure 2 illustrates the molecular models of NR and SBR, wherein “n” and “m” signify the number of recurring units within each molecule.

In the molecular modeling process, the NR content in the rubber component is maintained at 30%, while the SBR content is maintained at 70%. The number of repeat units in natural rubber is three, indicating its polymer structure. SBR, on the other hand, exhibits a chain length of 15. The composition of SBR is carefully controlled, with a styrene concentration of 23.5% and a butadiene concentration of 76.5%. The rubber content in rubber-modified asphalt constitutes about 12% of the total asphalt mass. The precise quantities of asphalt and rubber molecules are provided in Table 1.

### 2.2. Molecular Model Verification Parameter

To validate the model’s reliability, several properties of rubber-modified asphalt and neat asphalt were calculated and compared with experimental reference values. These properties included density, glass-transition temperature (Tg), cohesive energy density (CED), solubility parameter, and viscosity.

Density, a fundamental material property, holds significant importance in molecular model verification. In the molecular simulation of asphalt, the initial essential step involves determining the numerical value of density. The modeling process began with energy minimization using the “minimize” function to optimize the molecular structure. Subsequently, a 400 ps NVT simulation (constant number of particles (N), volume (V), and temperature (T)) was performed to reach a state of equilibrium. Following this equilibration, a 400 ps simulation was conducted using the NPT ensemble, where the pressure (P) was held constant to achieve the desired actual density. The NPT simulation consisted of an initial 100 ps phase with a pressure of 100 atm applied, followed by a subsequent 300 ps phase during which the pressure was gradually reduced to 1 atm. The choice of higher pressure at the early stage of the NPT ensemble was motivated by the fact that the model was initially established at a density of 0.1 g/cm^3^, and higher pressure expedited the convergence of density. These density simulations were conducted at 298.15 K (25 °C).

The glass transition temperature (Tg) marks the transition from the glassy to the rubbery state in asphalt. An excessively high Tg can lead to brittle asphalt prone to cracking, while an overly low Tg can cause asphalt to deform under loads and develop rutting. In molecular simulations, the glass transition temperature is determined by identifying abrupt changes in the temperature–density curve, which involves generating density results at various temperatures using the density simulation method for the asphalt model. By performing density simulations at intervals of 3 K within the temperature range of 100–400 K, density values corresponding to 100 temperatures were generated. The resulting scatter points were plotted to identify the range of the glass-transition temperature, which is commonly observed in asphalt between 233–282 K [17]. Overall, this procedure allowed for assessing the glass-transition temperature by repeatedly simulating density under different temperatures.

CED quantifies intermolecular interaction forces within a material, while the solubility parameter, equal to the square root of CED, assesses material compatibility. The formula for calculating CED is as follows:

(1) $$\mathrm{CED}=\left(\left[E_{\mathrm{total}}\right]-\left[E_{\mathrm{intra}}\right]\right)/V$$

where $E_{total}$ is the total energy of the asphalt system and $E_{intra}$ is the intramolecular energy of the asphalt molecules.

The formula for calculating solubility parameter δ is as follows:

(2) $$\delta =\sqrt{\mathrm{CED}}$$

Viscosity is a critical rheological property of asphalt, providing insights into its flow behavior. In this study, the asphalt viscosity was determined using the Green–Kubo formula [18]:

(3) $$\eta =\frac{V}{10k_{B}T}\int_{0}^{\infty }\;\langle \sum_{a,b}P_{\mathrm{ab}}{}^{\mathrm{st}}\left(0\right)P_{\mathrm{ab}}{}^{\mathrm{st}}(t)\rangle \mathrm{dt}$$

where η is the shear viscosity and V and T are the volume and temperature of the system, respectively. $k_{B}$ is the Boltzmann constant. $P_{\mathrm{ab}}{}^{\mathrm{st}}$ is the symmetrized traceless pressure tensor, and $∆A_{\mathrm{ab}}\left(t\right)=\int_{0}^{t}P_{\mathrm{ab}}{}^{\mathrm{st}}\left(t_{1}\right){\mathrm{dt}}_{1}$ indicates stress accumulation. The stress–tensor correlation function $\langle P_{\mathrm{ab}}{}^{\mathrm{st}}\left(0\right)P_{\mathrm{ab}}{}^{\mathrm{st}}\left(t\right)\rangle$ is used to improve convergence. The simulation was conducted at 408.15 K (135 °C) and ran for 12 ns with a correlation depth of 40 ps.

In the calculations conducted for this study, the CHARMM force field [19] was employed for the silica component, while the OPLS-aa force field [20] was utilized for the asphalt and rubber constituents. All molecular simulation calculations were conducted using the LAMMPS (Large-scale Atomic/Molecular Massively Parallel Simulator, v2022.04.07) software package [21].

**Figure 3:**
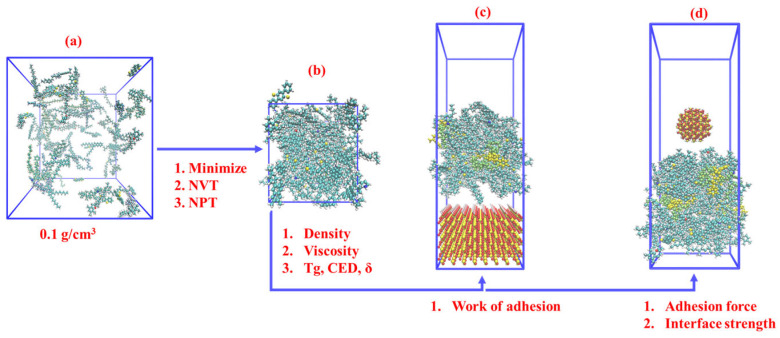
Simulation models and outcomes from each procedure, (**a**) initial asphalt model with a density of 0.1 g/cm^3^, (**b**) asphalt model with actual density, (**c**) silica-asphalt layer model, (**d**) silica-asphalt nanoindentation model.

Figure 3 provides an overview of this paper’s molecular simulation content. A density simulation is performed to replicate the asphalt model’s actual density accurately. Building upon this, simulations are conducted to investigate viscosity, Tg (glass transition temperature), CED, and solubility parameters. The molecular model of asphalt is constructed using Moltemplate software (v2023.02.06) [22], while the OPLS-aa force field [20] parameters for asphalt molecules are obtained from LigParGen software (v2.1) [23]. The specific modeling process involves synthesizing the coordinate information of asphalt molecules using Packmol software (v20.14.0) [24] and exporting the corresponding LT files for twelve distinct types of asphalt molecules through LigParGen software. Subsequently, Moltemplate software combines the PDB file synthesized by Packmol software with the LT file exported by LigParGen, resulting in a DATA file suitable for execution in LAMMPS. These modeling steps facilitate the initiation of simulations to study various parameters, including density, viscosity, and Tg.

Sandstone is used for experimental validation in this paper, where silica predominates as the primary constituent. For molecular modeling purposes, silica is chosen as a substitute for the aggregate in the simulations. To establish the silica supercell and spherical model with a radius of 10 Å, the CHARMM-GUI software (v1.9) [25] is employed, and the appropriate CHARMM force field [19] parameters are applied. Molecular coordinates are adjusted using Python code to ensure consistency with the coordinate system of asphalt molecules, thus enabling the successful merging of the silica–asphalt layered model. This merging process occurs within the Moltemplate software [22]. Figure 3c,d illustrates the resulting combined layered model, from which the work of adhesion, adhesion force, and interface stress can be determined.

The chip seal application can be configured with varying thicknesses, ranging from 37 mm to 9.5 mm, and may involve single or multiple layers. The aggregates utilized can also vary in size, spanning from 19 mm to 4.75 mm. In Figure 3d, the silica size measures 10 Å (1 Å = 1 × 10^−7^ mm), and the asphalt layer thickness is 35 Å, which aligns with the aggregate–asphalt thickness ratio of real-world chip seal practices. It is essential to note that this paper does not undertake an exhaustive investigation of the relative sizes of different silica and asphalt layers concerning their impact on adhesion performance.

### 2.3. Nanoindentation Simulation

This paper utilizes nanoindentation simulation to determine the adhesion force, as the silica indenter employed in the simulation penetrates the asphalt layer in a manner akin to the aggregate embedded within the asphalt layer in a chip seal. The molecular model of nanoindentation is shown in Figure 3d. To validate the findings of the nanoindentation analysis, this study conducted IBT experiments using rubber-modified asphalt and aggregates to assess the adhesion performance of rubber-modified asphalt on chip seal surfaces. The adhesion force derived from the nano-indentation unloading process closely resembles the pull-off effect observed in the IBT experiment.

The IBT experiment employed a tensile speed of 50 mm/min (millimeters per minute). In contrast, the unit of indentation speed (pull-off speed) utilized in the MD simulation is Å/fs (Angstrom per femtosecond). The relationship is given as 50 mm/min = 8.3333 × 10^−9^ Å/fs to establish an accurate conversion between the two units. This paper’s molecular simulation incorporates pull-off speeds of 0.001, 1 × 10^−4^, and 1 × 10^−5^ Å/fs. These specific values were selected based on practical considerations. As the pull-off speed decreases, the simulation duration increases significantly. Therefore, the choice of pull-off speed was influenced by the available computing resources and the desired computing time.

In nanoindentation simulation, this paper addresses the embedded depths of silica in the asphalt layer within the range of 1–20, 25, and 30 Å. For embedded depths falling within 1–20 Å, the curves are calculated in a continuous manner. This approach is necessitated by the silica indenter’s radius, which is set at 10 Å. By employing this methodology, the calculation range encompasses the conditions where the indenter first penetrates the asphalt layer and extends to a scenario where all of the indenter is embedded within the asphalt layer.

**Figure 4:**
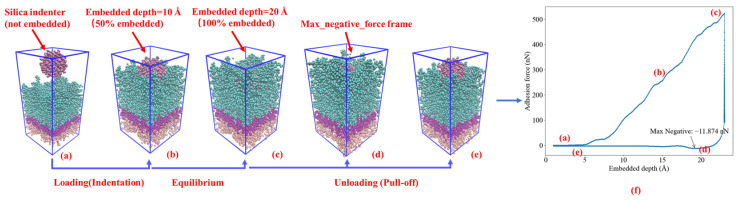
Schematic diagram of the simulation process of silica-asphalt nanoindentation, (**a**) initial silica–asphalt nanoindentation model, (**b**) silica embedded at 10 Å depth within an asphalt layer (50% of silica embedded), (**c**) silica embedded at 20 Å depth within an asphalt layer (100% of silica embedded), (**d**) the max negative force between silica and asphalt during pull-off process, (**e**) silica detached from the asphalt layer, (**f**) silica–asphalt nanoindentation curve with embedded depth of 20 Å and pull-off speed of 0.001 Å/fs.

Figure 4 illustrates the complete nanoindentation process, simulating the interaction between a silica indenter and an asphalt layer. The simulation comprises three main stages: loading, embedded depth maintenance, and unloading (pull-off). Initially, the silica indenter undergoes a loading process, exerting pressure on the asphalt layer at a defined rate. Subsequently, upon reaching the specified indentation depth, it achieves equilibrium, maintaining a balance for 50 ps. Following this, the unloading process commences, during which the silica indenter is gradually withdrawn from the asphalt layer at an identical rate as during the loading phase.

Figure 4 provides a comprehensive overview of the nanoindentation model at various critical points in the process. From left to right, it depicts the initial state of the nanoindentation (Figure 4a), the equilibrium states at embedded depths of 10 Å and 20 Å (Figure 4b,c), the frame where the maximum negative value occurs during unloading at a 20 Å embedded depth (Figure 4d), and, finally, a schematic representation of the silica indenter detached from the asphalt layer during the unloading process at 20 Å embedded depth (Figure 4e). The location corresponding to each frame of Figure 4a–e in the indentation curve is also indicated in Figure 4f.

Furthermore, it is essential to observe that the indentation model includes three distinct layers of color representing different functional regions within the asphalt layer. Proceeding from the bottom to the top, these layers are identified as the boundary, Thermostat, and Newtonian layers. The boundary layer remains fixed throughout the indentation process, effectively countering the effects of periodic boundary conditions. On the other hand, the Thermostat layer serves as a temperature control mechanism, ensuring that the Newtonian layer maintains a constant temperature of 298.15 K during the simulation. The Newtonian layer, governed by classical Newtonian mechanics, interacts with the silica indenter across the entire asphalt layer. This layer plays a pivotal role in the indentation process, providing insights into material behavior and responses under the influence of external forces.

**Figure 5:**
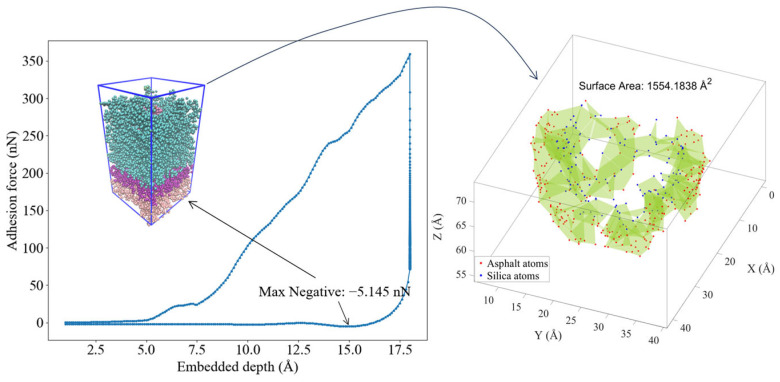
Adhesion Force–Indentation depth curve and surface area of Max-negative-value frame (indentation depth of 15 Å and speed of 0.001 Å/fs, rubber-modified asphalt).

Figure 5 depicts the indentation curve for the rubber-modified asphalt layer at an embedded depth of 15 Å and a speed of 0.001 Å/fs. The maximum negative value represents adhesion force between asphalt–silica during the unloading process. Determining the contact area is essential to assess the stress of the silica–asphalt contact interface where adhesion force is obtained. To achieve this, a MATLAB code was developed. The code’s primary approach involves categorizing all atoms in the selected frame into asphalt atoms and silica atoms. A critical criterion is applied: if the distance between any pair of asphalt atoms and silica atoms is less than 5 Å, these pairs are identified as being in mutual contact. This distance criterion is based on the typical effective range of van der Waals forces (around 3 to 5 Å) [26]. The atoms that pass this screening are then employed to construct a three-dimensional silica–asphalt contact surface. To accurately represent the contact area, the resulting area of the three-dimensional spherical surface is halved, considering its two sides (inner and outer surfaces).

Figure 5 also presents the results obtained from the MATLAB program, displaying a surface area of 1554.1838 Å^2^. The asphalt atoms are represented in red, while the silica atoms are depicted in blue. The constructed silica–asphalt contact surface resembles a spherical shape, aligning with extracting the spherical silica indenter from the asphalt atoms during the unloading phase. However, it is essential to acknowledge that at the molecular scale, there exist pores between molecules, leading to slight deviations from a perfect sphere.

Upon acquiring the adhesive force and its corresponding contact area, the stress of the contact surface can be calculated as follows: 5.145 nN/1554.1838 Å^2^ × 10^5^ = 331 MPa. This stress value offers valuable insights into the interaction and bonding between silica and asphalt, reflecting their mechanical behavior under specific loading conditions.

Figure 4 and Figure 5 provide a comprehensive overview of the methodologies employed in this study to determine the adhesion force and interface stress of the silica–asphalt contact interface using nanoindentation molecular simulation. Figure 5 focuses explicitly on investigating rubber-modified asphalt, utilizing an indentation depth of 15 Å and a speed of 0.001 Å/fs.

To ensure a comprehensive understanding of the simulation setup, Table 2 has been compiled, presenting a summary of all the critical parameters utilized in the nanoindentation calculations throughout this research. These parameters encompass various asphalt types, embedded depths, and pull-off speeds.

From a mechanical perspective, nanoindentation simulation offers valuable insights into the contact effect of silica–asphalt interfaces. In addition to mechanical analyses, the energy angle can further reinforce the observed contact effect. The model employed for this purpose is depicted in Figure 3c. The calculation is based on the following formula (modified from [27]):

(4) $$W_{\mathrm{adhesion}}=\frac{E_{\mathrm{interface}}-\left(E_{\mathrm{asphalt}}+E_{\mathrm{silica}}\right)}{A}$$

In this context, “$W_{\mathrm{adhesion}}$” represents the adhesion energy between silica and asphalt, while “$E_{\mathrm{interface}}$” denotes the total energy of the silica–asphalt interface system. Moreover, “$E_{\mathrm{asphalt}}$” represents the energy of the asphalt phase, and “$E_{\mathrm{silica}}$” signifies the energy of the silica phase. “A” denotes the contact surface area. A crucial observation is that the adhesion stress is directly proportional to the magnitude of the negative value obtained for the adhesion energy. Therefore, the greater the negative value, the higher the adhesion stress.

In addition to the total energy, this study quantifies the contributions of van der Waals energy (WvdW) and electrostatic potential energy (We). These energies serve as the primary components of non-bonding energy, and their calculated results offer valuable insights into the characteristics of the interface. By determining which energy plays a more dominant role and contributes more significantly to the interface, researchers could understand the fundamental forces at work within the system. Unless stated otherwise, properties in this article are calculated at 298.15K (25 °C).

### 2.4. Experiment Validation

To validate the findings of the nanoindentation analysis, this study conducted IBT experiments using rubber-modified asphalt and aggregates to assess the adhesion performance of rubber-modified asphalt in chip seal.

**Figure 6:**
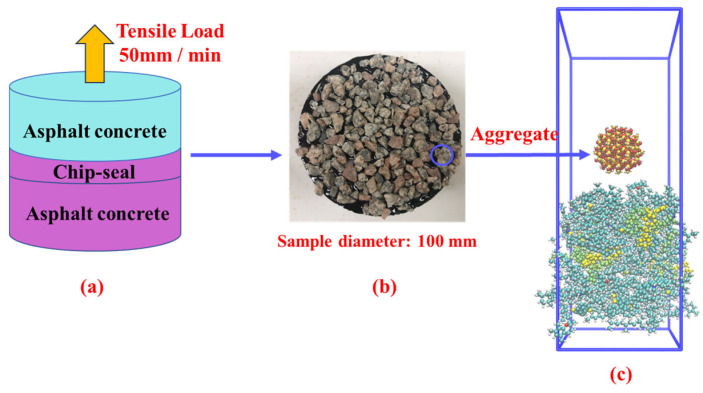
Chip seal sample IBT testing and modeling procedure, (**a**) pull-off test setting, (**b**) pull-offtest sample (chip seal part), (**c**) nanoindentation modeling of aggregate–asphalt layer contact.

This research used a lab chip seal to replicate the random distribution of spray aggregate on the pavement overlay. Figure 6 illustrates the lab chip seal sample. The sample was subjected to tension at a constant pull-off speed of 50 mm/min until fracture occurred. The maximum load applied during the test was recorded, and the interlaminar bond stress was calculated based on this data. The experimental samples’ specific preparation and experimental procedures can refer to the author’s previous papers [3].

The experimental samples were categorized into two groups: asphalt concrete layer and asphalt concrete layer with a chip seal layer. Figure 6b displays the arrangement of aggregate embedded within the asphalt layer. To investigate adhesion, nanoindentation simulation was employed in the corresponding molecular simulation to replace this embedded configuration, as depicted in Figure 6c. In the context of nanoindentation, the silica indenter is initially embedded within the asphalt layer, exhibiting a stable configuration, which mirrors the arrangement depicted in Figure 4b. The subsequent unloading process in nanoindentation entails extracting the silica indenter from the asphalt layer, reflecting a behavior corresponding to the pull-off effect observed in the IBT experiment.

## 3. Results

The results section comprises two main components. The first part presents the outcomes of model validation parameters, indicating the model’s adherence to a reasonable range and its agreement with experimental values. The second part focuses on the results of nanoindentation and work of adhesion, facilitating a comparison between the performance of rubber-modified asphalt and neat asphalt in chip seal scenery.

### 3.1. Molecular Model Verification

To validate the accuracy of the established asphalt molecular model, this study calculated various parameters, including density, CED, solubility, viscosity, and Tg. The calculated results are presented in Table 3.

Table 3 demonstrates that rubber-modified asphalt’s density is slightly lower than that of neat asphalt. Both density values fall within the reference range. This decrease in density can be attributed to the incorporation of rubber, a flexible and elastomeric material. Since rubber has a lower density than asphalt, its addition reduces the overall density of the mixture. It is worth noting that the rubber model in this study comprises 30% NR and 70% SBR. The molecular chain length and composition of rubber can influence the results. The validation performed in this study involves a general comparison of specific properties of the molecular model against reference values. The model is acceptable if the results are within the reference range.

The solubility parameters and CED of rubber-modified asphalt are slightly lower than those of neat asphalt. This is primarily because the solubility parameter of rubber typically ranges from 16 to 18 [32], while that of asphalt can range from 18 to 22.5 [29]. Upon mixing, the solubility parameter of rubber-modified asphalt decreases. This can be understood by employing the Hansen solubility parameter calculation method for mixtures, where the solubility parameters of the mixture are obtained by linearly combining the solubility parameters of each component and their respective proportions.

The viscosity of rubber-modified asphalt is slightly higher than that of neat asphalt, suggesting that the incorporation of rubber enhances intermolecular interactions within the binder, particularly at elevated temperatures. This increase in viscosity can be attributed to the elastomeric nature and higher molecular weight of rubber. The presence of rubber, a high-molecular-weight polymer, contributes to a stronger intermolecular network and, consequently, improves the high-temperature performance of the asphalt mixture.

The Tg of rubber-modified asphalt is lower than that of neat asphalt, which can be attributed to the inherent characteristics of rubber itself. Rubber is highly flexible and rubbery, while asphalt exhibits a more viscous or flowable state at elevated temperatures. For instance, the Tg range of NR typically falls between 203.15 K and 223.15 K [33]. Similarly, the Tg range of SBR is typically around 223.15 K to 243.15 K [34]. In contrast, neat asphalt typically has a higher Tg range, approximately 255 K and 282 K [17,31]. Consequently, incorporating rubber in asphalt decreases the Tg value, as rubber-modified asphalt exhibits a lower Tg compared to neat asphalt.

The above content highlights the analysis of model validation parameters, which closely match reference values and remain within a reasonable range. This substantiates the model’s credibility, rendering it suitable for further calculations. Subsequently, this section will delve into a comprehensive elucidation of the methodology employed to ascertain density, Tg (glass transition temperature), and viscosity.

**Figure 7:**
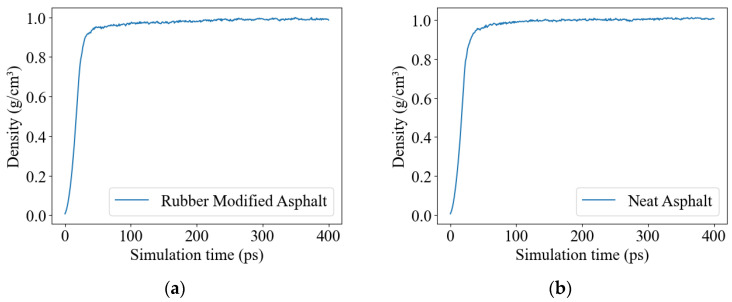
Density curve, (**a**) rubber-modified asphalt, (**b**) neat asphalt.

Figure 7 displays the density curves of rubber-modified asphalt and neat asphalt. To determine the density values in the steady state for each model, we calculate the average density within the time range of 300 ps to 400 ps.

It is important to note that determining the glass transition temperature encompasses a temperature range wherein the material experiences a significant transition in density. To ensure an objective selection of the glass transition temperature range, the author conducted a linear fitting analysis on 100 density data points acquired within the temperature range of 100 K to 400 K. Initially, a visual inspection suggested an estimated glass transition temperature range of 200 K to 300 K. Subsequently, the highest R^2^ values obtained from the linear fits in the low-temperature range (100 K to 300 K) and the high-temperature range (200 K to 400 K) were identified. The intersection of these two fitted lines closely approximates the glass transition temperature.

**Figure 8:**
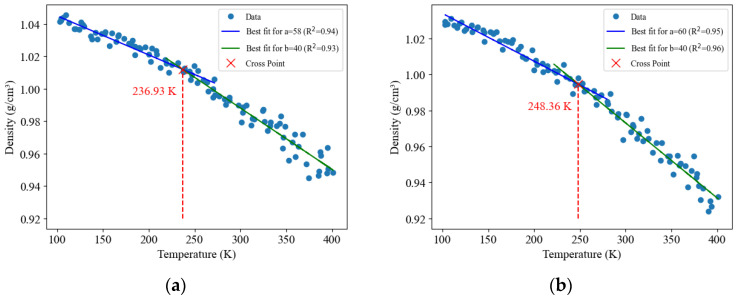
Glass transition temperature determination curve, (**a**) rubber-modified asphalt, (**b**) neat asphalt.

The parameter ‘a’ in Figure 8a indicates that the maximum R^2^ value was obtained using 58 data points from the low-temperature range (i.e., the first 58 data points out of the total 100), while ‘b’ in Figure 8a signifies that the maximum R^2^ value was obtained using 40 data points from the high-temperature range (i.e., the last 40 data points out of the total 100 data points).

**Figure 9:**
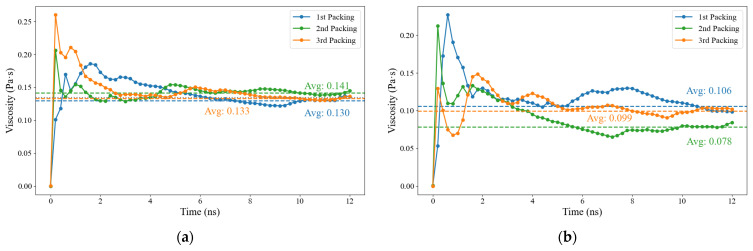
Viscosity determination curve, (**a**) rubber-modified asphalt, (**b**) neat asphalt.

Figure 9 displays the viscosity curves for rubber-modified asphalt and neat asphalt. Asphalt and rubber molecules are randomly positioned in the simulation cell using molecular modeling software. The figure presents the 1st, 2nd, and 3rd packing arrangements, involving the construction of the asphalt model thrice, to gauge the impact of random molecular distribution on viscosity. Analysis of these configurations reveals that the inherent randomness in molecular distribution exerts a negligible effect on viscosity outcomes. Notably, the viscosity curve exhibits discernible fluctuations during the initial stage. The viscosity values within the interval of 10 ns to 12 ns are averaged to determine a representative viscosity value. Additionally, the viscosity curves within this range demonstrate a tendency to converge. Achieving a smoother curve necessitates longer simulation times and larger correlation lengths, which, in turn, require more powerful computing resources. Specifically, in this study, calculating the viscosity of a single model with a correlation length of 40 ps consumed nearly 36 h on a 16-core CPU.

### 3.2. Silica-Asphalt Adhesion from Nanoindentation Simulation

#### 3.2.1. Embedded Depth and Pull-Off Speed Effect on Silica-Asphalt Adhesion

**Figure 10:**
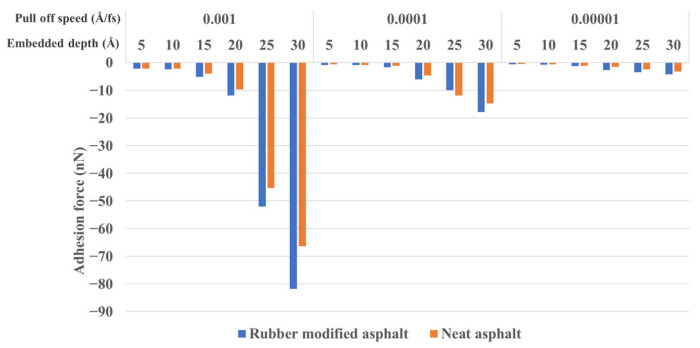
The adhesion force between silica and asphalt under different embedded depths and pull-off speeds.

Figure 10 presents the variations in adhesion forces between silica and asphalt during the nanoindentation unloading process, considering different embedded depths and pull-off speeds. The adhesion force is determined by the maximum negative value of the curve obtained during the nanoindentation’s unloading phase, as explained in Section 2.3.

The analysis of Figure 10 reveals notable trends. Firstly, a decrease in pull-off speed correlates with a decrease in adhesion between silica and asphalt. Conversely, an increase in embedded depth leads to higher adhesion forces. During the initial penetration phase of the indenter (embedded depth from 5–10 Å), the influence of the pull-off speed on adhesion appears to be minimal. However, the adhesion force prominently increases with higher pull-off speeds from half to complete embedding. Moreover, a significant growth in adhesion is observed after the silica indenter has fully penetrated the asphalt layer at the same pull-off speed.

Furthermore, the results demonstrate that, compared to neat asphalt, rubber-modified asphalt exhibits superior adhesion to silica in most scenarios. This molecular simulation perspective provides valuable insights into the favorable performance of rubber-modified asphalt for chip seal applications.

**Figure 11:**
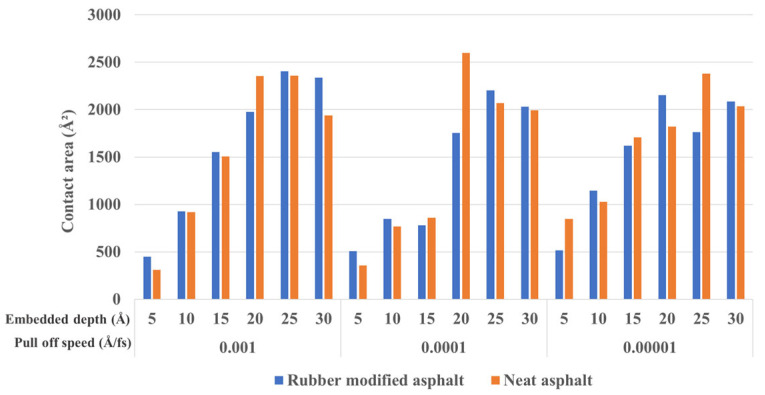
The contact area between silica and asphalt under different embedded depths and pull-off speeds.

Figure 11 provides insight into the contact area between silica and asphalt at the state corresponding to the maximum negative adhesion force (observed during unloading) for various embedded depths and pull-off speeds. As depicted in Figure 11, the pull-off speed does not significantly influence the contact area between silica and asphalt. Conversely, a deeper indentation depth corresponds to a larger contact area during the unloading process, indicating enhanced adhesion. Moreover, the impact of rubber-modified and neat asphalt on the contact area does not exhibit distinct regular patterns, as illustrated by the data.

**Figure 12:**
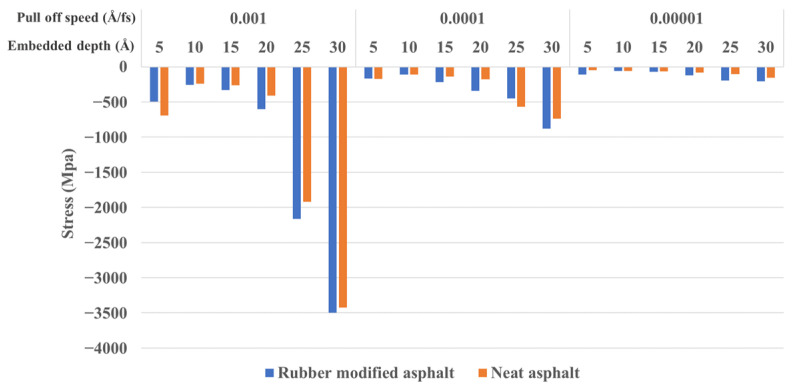
Interface stress between silica and asphalt under different embedded depths and pull-off speeds.

Figure 12 depicts the stress values of the silica-asphalt contact surface, representing the strength of the interface, with the adhesion force during the unloading process for varying embedded depths and pull-off speeds. The results demonstrate a significant impact of the pull-off speed on the contact surface stress, with a clear trend of lower velocities resulting in reduced interface stress. The lowest contact stress is observed at a pull-off speed of 1 × 10^−5^ Å/fs. Additionally, deeper embedded depths correspond to higher contact surface stress. Moreover, the contact surface stress of rubber-modified asphalt surpasses that of neat asphalt.

**Figure 13:**
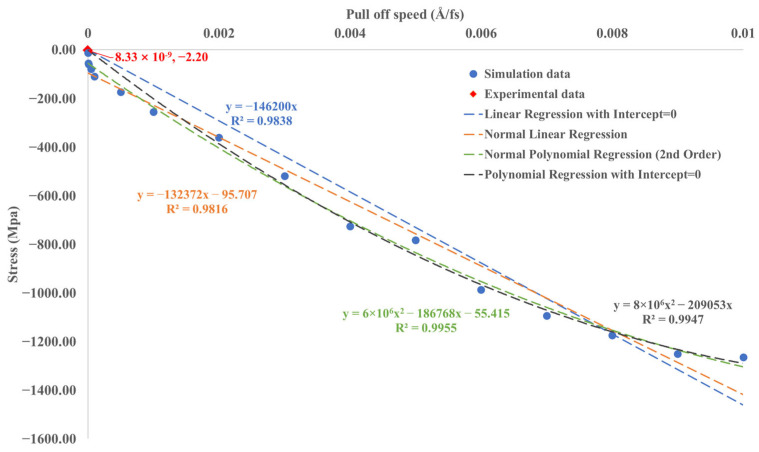
Stress between silica and rubber-modified asphalt under different pull-off speeds (embedded depth = 10 Å).

Figure 13 illustrates the interface stress between silica-rubber modified asphalt under varying pull-off speeds, with an embedded depth of 10 Å, as well as the experimental data. A 10 Å embedding depth was chosen to match the approximate 50% embedding degree of aggregate particles within the asphalt layer in the experiment. This depth also provides computational efficiency for calculating small pull-off speeds compared to larger embedding depths. It is worth mentioning that the experimental contact surface stress, approximately 2.2 MPa, is lower than the simulated value, which can be attributed to the variation in pull-off speeds. The experimental pull-off speed is 8.3333 × 10^−9^ Å/fs, while the simulated pull-off speed range is 1 × 10^−7^ Å/fs–0.01 Å/fs. Employing smaller indentation speeds can yield similar results to the experiment but requires additional simulation time. This study attempted a smaller pull-off speed of 1 × 10^−7^ Å/fs and an embedded depth of 10 Å for rubber-modified asphalt, but the model necessitated a calculation time of 15 days.

The scatter plot represents the data used for fitting, which include indentation speeds of 0.005, 5 × 10^−4^, 5 × 10^−5^, 5 × 10^−6^, 0.002, 0.003, 0.004, 0.006, 0.007, 0.008, 0.009, 1 × 10^−7^ Å/fs, in addition to the values of 0.001, 1 × 10^−4^, and 1 × 10^−5^ Å/fs shown in Figure 13.

The findings of this study demonstrate a high degree of agreement between the linear and quadratic fits for simulation data, yielding an impressive R^2^ value over 98%. The silica–asphalt interface stress reduces as the pull-off speed decreases, gradually converging toward the experimental data point. The graphical representation illustrates the proximity of linear and quadratic regression lines with intercept = 0 to the experimental data (red diamond shape). When incorporating the experimental pull-off speed (8.33 × 10^−9^ Å/fs) into the four regression models, the corresponding stress values are approximately −0.0012 MPa (linear regression with intercept = 0), −95.708 MPa (standard linear regression), −55.417 MPa (standard polynomial regression), and −0.0017 MPa (polynomial regression with intercept = 0). These calculated values exhibit an order of magnitude difference compared to the experimentally obtained stress, which is influenced by the choice of regression method. At a minimum pull-off speed of 1 × 10^−7^ Å/fs used in MD simulation, the stress is −12.159 MPa, closely matching the experimental results’ order of magnitude. However, attaining even smaller pull-off speeds for accurate simulation–experiment comparisons exceeds current computational capabilities. To bridge the gap between simulated and experimental pull-off speeds while maintaining similar contact surface stress, a potential solution involves utilizing GPU-accelerated calculations to establish a quantitative regression relationship between different pull-off speeds and contact surface stress, which will be studied in the future. Notwithstanding the variation in pull-off speed employed in this simulation compared to the experimental conditions, the resultant outcomes remain comparable. Such a configuration is also evident in prior molecular dynamics (MD) simulation investigations [28,35].

#### 3.2.2. Silica–Asphalt Adhesion Simulated under Continuous Embedded Depth

In this section, the primary objective is to investigate the impact of the continuous embedded depth of the silica indenter, specifically its transition from entering the asphalt layer to fully immersing in it, on the adhesion between silica and asphalt. To achieve this, the indentation depth varies from 1 Å to 20 Å, with the indenter’s radius set at 10 Å. It is important to note that the pull-off speed employed in this section is 1 × 10^−5^ Å/fs.

**Figure 14:**
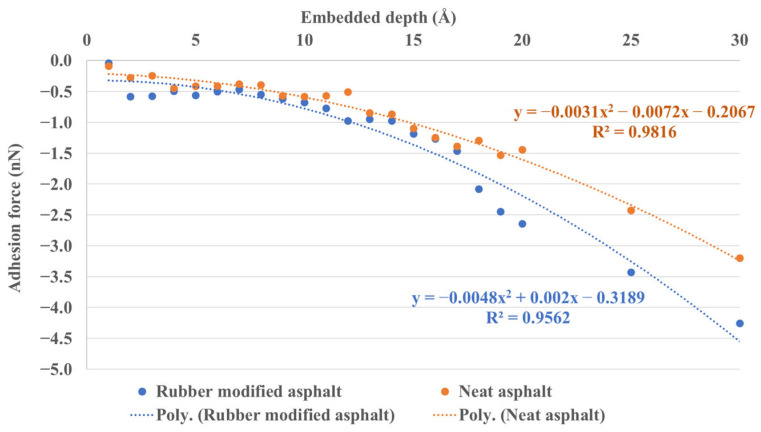
The adhesion force between silica and asphalt under continuous embedded depth.

Figure 14 displays the adhesion force between silica and asphalt for a range of continuous embedded depths from 1 Å to 20 Å, including results at 25 Å and 30 Å. It is evident from the graph that the adhesion performance of rubber-modified asphalt to silica during unloading (pulling off) is consistently superior to that of neat asphalt. Moreover, as the indentation depth increases, the adhesion force also exhibits a corresponding increase, following a one-dimensional quadratic equation pattern. This observation can be rationalized by considering that deeper embedded depths lead to increased atomic interactions between the materials.

**Figure 15:**
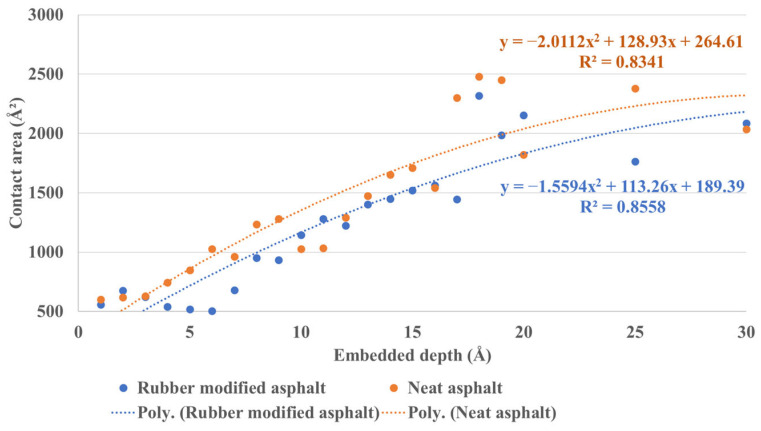
The contact area between silica and asphalt under continuous embedded depth.

Figure 15 illustrates the contact area between silica and asphalt under a series of continuous embedded depths ranging from 1 Å to 20 Å and includes the result of 25 Å and 30 Å. A clear trend demonstrates that the contact area increases with the embedded depth. Furthermore, the contact area between rubber-modified asphalt and the silica indenter is smaller overall than neat asphalt. When considering the specific criterion of 5 Å for contact area evaluation (as outlined in Section 2.3), it becomes apparent that the number of silica atoms–asphalt atoms pairs meeting the distance condition in rubber-modified asphalt is relatively fewer than in neat asphalt. This phenomenon can be attributed to the lower density of rubber-modified asphalt, leading to an increased presence of pores under comparable conditions. Consequently, rubber-modified asphalt generally exhibits a smaller contact area under the same data processing method.

**Figure 16:**
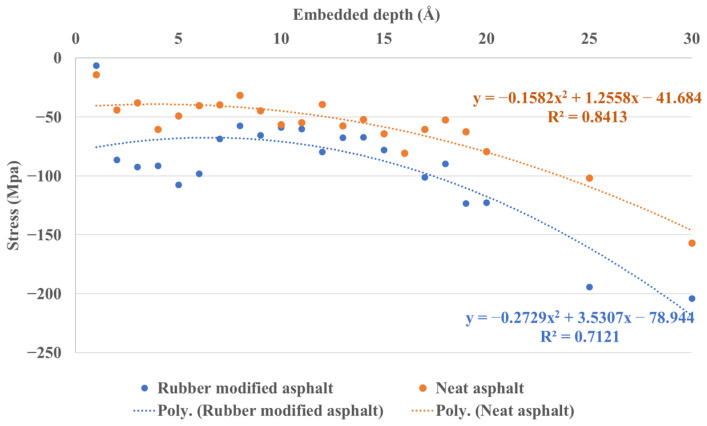
Stress between silica and asphalt under continuous embedded depth.

Figure 16 depicts the interface stress between silica and asphalt at various continuous embedded depths, ranging from 1 Å to 20 Å, and includes additional data points at 25 Å and 30 Å. The findings reveal that, overall, the interface stress between rubber-modified asphalt and silica surpasses that of neat asphalt. Moreover, the interfacial stress of both asphalt and silica increases with increasing embedded depth. However, it is noteworthy that the trend-fitting results based on the quadratic equation show a less-than-ideal fit. A larger data set would facilitate accurate analyses to achieve more precise trend-fitting outcomes.

#### 3.2.3. Work of Adhesion between Asphalt and Silica

**Figure 17:**
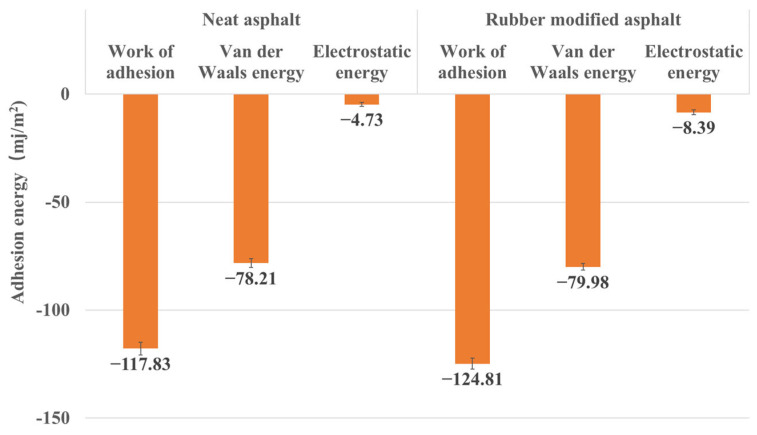
Work of adhesion between asphalt and silica.

Figure 17 presents the adhesion energy between rubber-modified asphalt, neat asphalt, and silica. The figure indicates that the adhesion between rubber-modified asphalt and silica is stronger. This observation aligns consistently with the results obtained from nanoindentation simulations. The viscosity increases upon incorporating rubber into the asphalt mixture, leading to stronger intermolecular forces and enhanced silica adhesion. Additionally, the figure reveals an amplification in the electrostatic potential energy within intermolecular interactions after rubber incorporation, contributing to the overall interfacial energy. Nonetheless, it is noteworthy that van der Waals forces continue to exert the primary influence in these interactions.

## 4. Discussion

This paper explores the molecular-level applications of neat asphalt and rubber-modified asphalt in chip seal, primarily focusing on the interaction between aggregate and asphalt layers. Energy-based (work of adhesion) and mechanical-based (adhesion force and interface stress from pull-off simulations) analyses consistently provide valuable insights. The study highlights that interfacial adhesion between aggregate and asphalt is influenced by asphalt type, aggregate embedding depth, and pull-off speed. Notably, the investigation considers explicitly a rubber content of approximately 12% and examines the effects of pull-off speed and embedding depth. However, chip seal applications are multifaceted, encompassing factors such as varying asphalt sources (with differing SARA ratios), temperature, moisture, aggregate type, aggregate roughness, and environmental conditions (including acidity, alkalinity, and salt exposure), all of which can impact outcomes. These variables will be the focus of future research endeavors.

A notable observation is the convergence of simulated interface stress with experimental results as the pull-off speed decreases. Specifically, at the minimum pull-off speed of 1 × 10^−7^ Å/fs employed in simulations, the interface stress reaches 12.159 MPa, a value closely mirroring the order of magnitude of the interface stress recorded during experimental testing at a pull-off speed of 8.3333 × 10^−9^ Å/fs (2.2 MPa). It is essential to acknowledge that further reducing the pull-off speed was impeded by computational limitations. The accelerated implementation of GPU computing is under consideration to overcome this constraint. Subsequent research endeavors will focus on corroborating the consistency between molecular simulations and experimental results and strive to establish a quantitative relationship for interface stress under various pull-off speeds. If such a relationship can be established, it may facilitate result conversion between experiments and simulations, even when different pull-off speeds are employed.

## 5. Conclusions

This paper examines the molecular scale scenarios of rubber-modified and neat asphalt in chip seal application. The asphalt molecular model’s reliability was validated using various parameters, including density, viscosity, cohesive energy density, solubility, and glass transition temperature. Furthermore, the nanoindentation and silica–asphalt interface models were established to calculate the adhesion force, interface stress, and work of adhesion. The obtained conclusions are as follows:

1. Rubber-modified asphalt exhibits slightly lower density, cohesive energy density, and solubility parameters and obviously lower glass transition temperature compared to neat asphalt, which is attributed to the inclusion of rubber. Conversely, the viscosity of rubber-modified asphalt is slightly higher than that of neat asphalt.
2. In the nanoindentation model, the adhesion force between silica and asphalt increases proportionally with the embedded depth and pull-off speed. The contact area of the silica-asphalt corresponding to the maximum adhesion force increases with the embedded depth while showing no significant correlation with the pull-off speed. Furthermore, the silica–asphalt interface stress displays an increment with both embedded depth and pull-off rate. This indicates that deeper embedded aggregates in the asphalt layer exhibit stronger adhesion at the molecular level in the chip seal application.
3. The simulation results based on continuous embedded depth reveal a quadratic regression relationship between adhesion force, interface stress, and indentation depth.
4. The work of adhesion results demonstrate that the adhesion between rubber-modified asphalt and silica is slightly stronger than that between neat asphalt and silica, thereby validating the concordance between energy (work of adhesion) and mechanical outcomes (adhesion force).

Overall, these molecular-scale findings shed light on the improved adhesive properties and applicability of rubber-modified asphalt compared to neat asphalt in chip seal applications.

## Figures and Tables

**Table 1:** Molecules in asphalt model (asphalt molecule data from Greenfield [12]).

	Component	Number of Molecules	Weight %
Saturate	Squalane	4	9.83
	Hopane	4	
Aromatic	Perhydrophenanthrene-naphthalene	11	28.22
	Dioctyl-cyclohexane-naphthalene	13	
Resin	Quinolinohopane	4	35.15
	Thioisorenieratane	4	
	Trimethylbenzeneoxane	5	
	Pyridinohopane	4	
	Benzobisbenzothiophene	15	
Asphaltene	Asphaltene-phenol	3	15.19
	Asphaltene-pyrrole	2	
	Asphaltene-thiophene	3	
Rubber	NR (natural rubber)	6	11.61
	SBR (styrene butadiene rubber)	3	

**Table 2:** Summary of nanoindentation simulation setup.

		Rubber Modified Asphalt	Neat Asphalt
		Embedded Depth (Å)	
Pull-off speed (Å/fs)	0.001	5, 10, 15, 20, 25, 30	5, 10, 15, 20, 25, 30
	1 × 10^−4^	5, 10, 15, 20, 25, 30	5, 10, 15, 20, 25, 30
	1 × 10^−5^	1–20, 25, 30	1–20, 25, 30
	1 × 10^−6^	15	/

**Table 3:** Properties for asphalt from molecular simulations.

	Rubber Modified Asphalt	References	Neat Asphalt	References
Density (g/cm^3^)	0.99	/	1.01	0.99–1.15 [11,28]
Cohesive energy density (J/m^3^)	3.95 × 10^8^	/	3.98 × 10^8^	3.19–4.00 × 10^8^ [11,29]
Solubility parameter (J/cm^3^)^0.5^	19.88	/	19.94	18–22.5 [11,29]
Viscosity (Pa·s, 408.15 K)	0.135	0.1–3.6 [13,30]	0.095	0.06–0.12 [13,30]
Glass transition temperature Tg (K)	Around 236.93	233–255 [17]	Around 248.36	255–282 [17,31]

## Data Availability

Some or all data, models, or codes that support the findings of this study are available from the corresponding author upon reasonable request.
